# Idiopathic superficial siderosis of the central nervous system

**DOI:** 10.1186/s40673-021-00133-5

**Published:** 2021-02-25

**Authors:** Shakila Meshkat, Parnia Ebrahimi, Abbas Tafakhori, Aidin Taghiloo, Sajad Shafiee, Amir Salimi, Vajiheh Aghamollaii

**Affiliations:** 1 Faculty of Medicine, Tehran University of Medical Sciences, Tehran, Iran; 2 Department of Neurology, Roozbeh Psychiatric Hospital, Tehran University of Medical Sciences, Tehran, Iran; 3 Department of Psychiatry, Roozbeh Psychiatric Hospital, Tehran University of Medical Sciences, Tehran, Iran; 4 Iranian Center of Neurological Research (ICNR), Tehran University of Medical Sciences, Tehran, Iran; 5 Department of Radiology, Tehran University of Medical Sciences, Tehran, Iran; 6 Department of Neurosurgery, Mazandaran University of Medical Sciences, Sari, Iran; 7 Faculty of Medicine, Shahid Beheshti University of Medical Sciences, Tehran, Iran

**Keywords:** Chronic subarachnoid hemorrhage, Hemosiderin, Sensorineural deafness, Superficial siderosis

## Abstract

**Background:**

Regardless of the cause of the superficial siderosis (SS) disease, which is bleeding, the source of bleeding cannot be found in some cases.

**Case presentation:**

In this article, we report two cases with idiopathic SS. Case 1 presented with bilateral hearing loss, cognitive impairment, sleep disturbances, and tremors. Case 2 presented with sensory neural hearing loss, ataxia, and spastic paraparesis. In both cases, brain MRI indicated evidence of SS. CT myelogram and SPECT with labeled RBC couldn’t help finding the source of occult bleeding.

**Conclusion:**

SS is a rare central nervous system disease caused by the deposition of hemosiderin in the brain and spinal cord, which results in the progression of neurological deficits. The cause of this hemorrhage is often subarachnoid haemorrhage, intracranial surgery, carcinoma, arteriovenous malformation, nerve root avulsion, and dural abnormality. The condition progresses slowly and, by the time diagnosis is confirmed, the damage is often irreversible. In our cases, brain MRI clarified the definitive diagnosis, but we could not find the source of bleeding. SS should be considered in cases with ataxia and hearing loss, even if no source of bleeding is found.

## Introduction

Superficial siderosis (SS) is an extremely rare central nervous system (CNS) condition in which hemosiderin (a product of the breakdown of blood) is deposited in the leptomeninges, subpial layer, ependymal surface, and other parts of the CNS and results in progressive neurological dysfunction [1]. SS affects all ages and both sexes. The prevalence in the general population is unclear; however, studies have reported a range of 0.21–1.43% in the population older than 55 years, rising in the population over 69 years [2]. The clinical manifestation is characterized by a triad of sensorineural hearing loss, cerebellar ataxia, and myelopathy, accompanied by dementia and sphincter disturbances. SS was first described 100 years ago, and the diagnosis was based on an autopsy before the widespread use of magnetic resonance imaging (MRI) [3]. SS’s etiology is reported to be a chronic subarachnoid hemorrhage originating from an occult source in most cases. While the other cases are secondary to a known cause of subarachnoid hemorrhage such as a CNS tumor, arteriovenous malformation (AVM), or trauma, however, some cases are idiopathic [4].

Given the unspecific sensorineural, neurological, and cerebrovascular findings associated with SS combined with its low population prevalence, the condition can often be misdiagnosed or entirely missed. In this paper, we describe diagnostic and treatment considerations in two patients with SS.

## Case reports

### Case 1

**Fig. 1:**
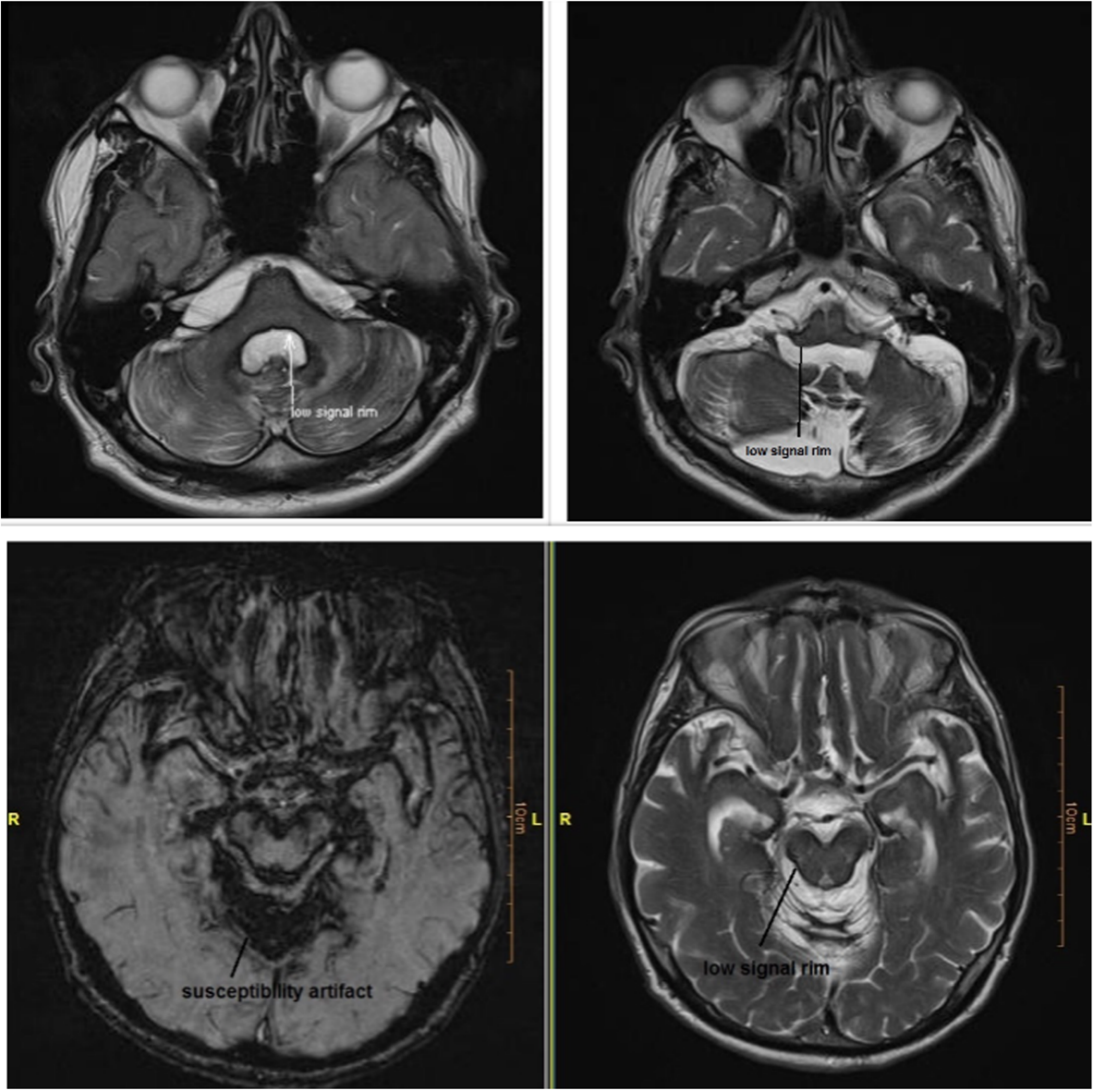
MRI brain images of the patient 1 showing features characteristic of superficial siderosis. 1. Low rim coating the surface of the brain and brain stem particularity cerebellum with obvious signal void intensity. 2. High signal intense rim in T1 sequences is coated brain stem and cerebellar vermis

A sixty-two-year-old man presented to our neurology clinic with complete bilateral hearing loss, which began at the age of thirty-five. Twelve years ago, he started feeling unsteadiness in his lower limb, especially in the left one, which was slowly progressive, and now although he could still stand, he suffered from gait imbalance and could not walk without help. The patient was referred to the local county hospital, but the treatment was unsuccessful. Gradually the symptoms worsened, and when the patient presented at our clinic, he could not walk and do daily livings independently. He also experienced cognitive impairment, sleep disturbances, tremors, and difficulty in swallowing since two years ago. He had no past medical history. He did not have a history of falling or accidents. He declined to undergo any severe headache or brain / spinal surgery. He had a negative family history. The general physical examination was normal. Neurological examination revealed bilateral hearing loss (air conduction > bone conduction), reduced muscle force of the lower limbs, bilateral Babinski sign, bilateral pain and temperature loss below T3, weak finger to nose test, poor heel shin test, and moderate dysdiadochokinesia. He had abnormal mental status with memory impairment, and reflexes were increased. Mini-mental state examination (MMSE) score was 21 (delayed recall score:0). Laboratory analysis findings, including blood tests, urine tests, liver function tests, renal function tests, blood electrolytes, coagulation, thyroid function tests, folic acid, B12, ferritin, etc, were all within the normal range. He underwent brain MRI (1.5 T), which indicated low signal rim coating the surface of the brain and brain stem, particularly the cerebellum, with obvious signal void intensity in susceptibility weighted imaging (SWI) sequences (Fig. 1). Computed tomography (CT) myelogram and single photon emission CT (SPECT) with labeled red blood cell (RBC) were negative. SS diagnosis was made based on neuroimaging, and treatment started with an iron-chelating agent (deferiprone). He was also given Rivastigmine for cognitive impairment. Cochlear implantation was recommended for his hearing loss. At follow-up visit after 6 months, he reported complete recovery in swallowing, tremor, and some improvement in gait imbalance. However, follow-up brain MRI showed no obvious changes in comparison to the previous one. After 1 year, we tapered Rivastigmine, as his cognitive function improved dramatically (MMSE: 26), and after 18 months, when neurocognitive evaluations revealed normal cognitive function, Rivastigmine discontinued.

### Case 2

**Fig. 2:**
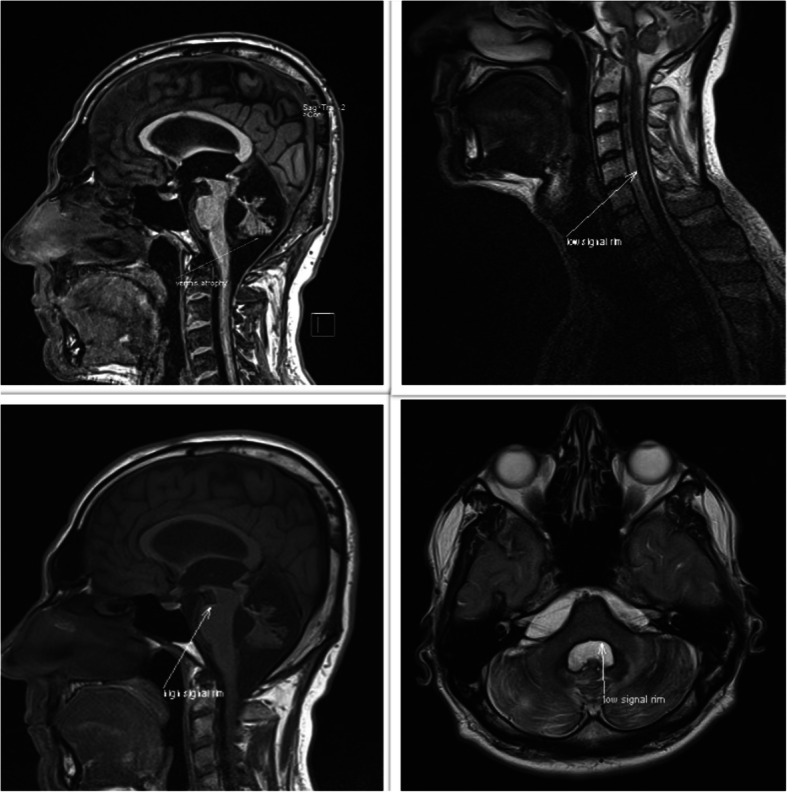
MRI brain images of the patient 2 showing features characteristic of superficial siderosis. A rim of low intensity coating the surface of the brain and spinal cord with atrophy of cerebellar vermis

A thirty-year-old man was referred to our neurology clinic with a slow progression of sensory neural hearing loss, ataxia, and spastic paraparesis. His symptoms started seven years ago and got worsened during the course of the disease. He also experienced severe headaches and was admitted to our neurosurgery department with hydrocephalus diagnosis and underwent shunting. He had mild sphincter dysfunction but no erectile dysfunction. The patient denied any olfactory and visual impairment, memory loss, seizures, and loss of consciousness. There was no history of trauma, surgery, and CNS tumor. There was no evident history of intracranial hemorrhage. He didn’t report any past intracranial or spinal surgery. There was no family history of similar conditions. The general physical examination was normal. Neurological examination revealed bilateral hearing loss (air conduction > bone conduction), bilateral Babinski sign, dysarthria spastic quadriparesis, and limb ataxia. There was no sensory problem. His mental state was normal, with no cognitive impairment. The MMSE score was 28. Laboratory analysis, including blood tests, urine tests, liver function tests, renal function tests, blood electrolytes, coagulation, thyroid function tests, folic acid, B12, ferritin, etc, were all within the normal range. He underwent brain MRI, which indicated low signal intensity coating the surface of the brain and spinal cord and atrophy of cerebellar vermis (Fig. 2). CT myelogram and SPECT with labeled RBC couldn’t help finding the source of occult bleeding. SS diagnosis was made based on neuroimaging, and he was given deferiprone as treatment. After 18 months of initiation of the iron chelator, there was a modest recovery in gait and ataxia, but no obvious changes in the follow-up brain MRI were seen compared to the previous one.

## Discussion

SS is a rare disease characterized by cerebellar dysfunction, hearing loss, and spastic paresis. Other symptoms can be dysphagia, bladder disturbance, dementia, anisocoria, and anosmia. Besides, cerebellar ataxia and deafness occur in 90% of cases [4]. In our study, both patients presented with signs of cerebellar dysfunction, gait imbalance, progressive sensorineural hearing loss, and dementia. SS is mostly caused by chronic or recurrent bleeding. The most common causes of bleeding are head or neck trauma, AVM, CNS tumors, previously CNS surgery, brachial plexus injury, and other cause of subarachnoid hemorrhage. Moreover, cerebral amyloid angiopathy is a cause of SS in older individuals. However, about 35% of SS cases are idiopathic [5, 6]. As in our study, the source of bleeding in both patients was not identified. Both patients did not have a history of previous trauma, and they never exhibited any intense intracranial hemorrhage symptoms. Table 1 shows clinical characteristics of reported cases with idiopathic SS [7–14].

**Table 1:** Clinical characteristics of reported cases with idiopathic SS

Author	Gender	Age (years)	Symptoms duration (years)	Reported symptoms
Chen et al	Male	62	1.5	Slurred speech and ataxia
Stabile et al	Male	45	8	Progressive gait difficulties, tinnitus and emotional liability
Vale et al	Male	49	6	Cerebellar ataxia, bilateral auditory loss, mild cognitive impairment
Matsuyama et al	Male	59	4	Cerebellar ataxia, hearing loss and disturbance of memory
Posti et al	1 Female 1 Male	F: 66 M: 59	F: 1 M:1	F: Severe headache, photophobia, meningismus, and nausea M:Unconsciousness and hemiparesis
Nakane et al	Female	57	Not reported	Sensorineural hearing impairment, cerebellar ataxia, pyramidal tract signs, dysarthria, and neurogenic bladder
Phanthumchinda et al	Male	58	5	Chronic progressive hearing loss and gait instability
Kresojević et al.	Female	71	7	Progressive bilateral hearing loss, unsteady gate, hand clumsiness and dysarthria

The mechanism of SS is still unknown; however, the breakdown of erythrocytes or RBCs from blood results in lysing and hemosiderin release. In reaction to this increase in heme levels, heme oxygenase-I enzyme is released by Bergmann glia and microglia cells, which splits the free heme into biliverdin and carbon monoxide iron [15, 16]. The deposition of hemosiderin is associated with demyelination, gliosis, and neuronal loss. Sensorineural hearing loss is the most common symptom of SS that leads to complete deafness in 1 to 12 years [17]. In our study, the first case developed complete deafness after 12 years at the age of thirty-five.

The diagnosis of SS was based on autopsies and biopsies before the use of neuroimaging. SS can also be diagnosed by cerebrospinal fluid (CSF) examination through xanthochromia, elevated presence of RBCs, high iron and ferritin concentrations, and elevated levels of the proteins Tau, beta-amyloid (Ab42), and glial fibrillary acidic protein (GFAP), but in most of the cases CSF is normal [18]. CT scan of SS patients shows high signal rims and/or atrophy. SS-CNS is currently diagnosed by MR images demonstrating pathological findings caused by iron deposition beneath the surface of CSF in CNS tissues [7]. The characteristic pathological finding is the existence of a signal loss on the surface of the CNS structures on a T2-weighted image. Cerebellar atrophy, spinal cord atrophy, and hyperintense rime can also be seen. SWI is a new MR sequence that exhibits extreme sensitivity to blood-degradation products i.e. iron and hemosiderin. For the first time in Iran, SPECT for Tc-99 m labeled RBC for detecting hemosiderin was performed in both patients but revealed nothing in favor of occult bleeding source. Finally, in our study, both patients underwent SWI MRI, and SS were confirmed based on MRI findings [1, 15, 19].

SS is a progressive neurological condition that must be identified early in order to be successfully treated. Studies have indicated that the first presentation usually appears after at least 6 months of subarachnoid hemorrhage from injury in 97% of cases [17, 20]. When the source of hemorrhage is detected in MRI, the purpose of treatment should be to discourage the disease’s worsening by eliminating the source of subarachnoid hemorrhage surgically. Unfortunately, there is no treatment to reverse the hemosiderin deposition damaging effects [21]. The most common therapy is iron chelators, such as deferiprone and desferrioxamine. Among iron chelators, deferiprone can cross the blood-brain-barrier to chelate the hemosiderin in the CNS [6]. In a clinical trial by Levy et al, ten patients with SS were treated with deferiprone, and after 90 days of treatment, four patients revealed hemosiderin deposition decrease on MRI [22]. Another study by Kessler et al, on 38 patients with SS demonstrated a measurable reduction in hemosiderin by MRI in half of patients after 2 years of follow up. Also, the safety profile of deferiprone was confirmed [23]. Although, most of the reported cases did not report significant improvement in symptoms or halting the disease progression using iron chelators [20]. In our study, case 1 indicated the complete recovery of cognitive function and improvement of other symptoms to a certain extent. But, case 2 represented modest recovery in gait disturbance, ataxia, and hearing loss. However, none of patients had reduction in hemosiderin in their follow up brain MRI.

In conclusion, SS is a rare CNS disorder caused by hemosiderin deposition in the leptomeninges, subpial layer, ependymal surface and can result in neurological dysfunction and progressive, irreversible signs and symptoms. Here, we presented two cases of SS with severe symptoms that were misdiagnosed. To prevent potential misdiagnoses, we recommend that SS should be considered as a clinical diagnosis for patients, particularly those on anticoagulation and with or without a history of brain damage or accident, which have bilateral sensorineural hearing loss and gait ataxia.

## Data Availability

The datas used for this study are available from the corresponding author on reasonable request.
